# Do Androgens Modulate the Pathophysiological Pathways of Inflammation? Appraising the Contemporary Evidence

**DOI:** 10.3390/jcm7120549

**Published:** 2018-12-14

**Authors:** Abdulmaged Traish, Jose Bolanos, Sunil Nair, Farid Saad, Abraham Morgentaler

**Affiliations:** 1 Department of Urology, Boston University School of Medicine, Boston, MA 02118, USA; 2 Men’s Health Boston, Chestnut Hill, MA 02467, USA; jose@menshealthboston.com; 3 Division of Pulmonary and Critical Care Medicine, Department of Medicine, New York University School of Medicine, New York, NY 10016, USA; ssnair84@gmail.com; 4 Medical Affairs Andrology, Bayer AG, 13353 Berlin, Germany; farid.saad@bayer.com; 5 Research Department, Gulf Medical University School of Medicine, Ajman, UAE; 6 Men’s Health Boston, Beth Israel Deaconess Medical Center, Harvard Medical School, Chestnut Hill, MA 02467, USA; amorgent@yahoo.com

**Keywords:** testosterone, C-reactive protein, tumor necrosis factor-alpha, interleukin-6, interleukin-1 beta, chronic inflammatory diseases

## Abstract

The role of testosterone in the pathophysiology of inflammation is of critical clinical importance; however, no universal mechanism(s) has been advanced to explain the complex and interwoven pathways of androgens in the attenuation of the inflammatory processes. PubMed and EMBASE searches were performed, including the following key words: “testosterone”, “androgens”, “inflammatory cytokines”, “inflammatory biomarkers” with focus on clinical studies as well as basic scientific studies in human and animal models. Significant benefits of testosterone therapy in ameliorating or attenuating the symptoms of several chronic inflammatory diseases were reported. Because anti–tumor necrosis factor therapy is the mainstay for the treatment of moderate-to-severe inflammatory bowel disease; including Crohn’s disease and ulcerative colitis, and because testosterone therapy in hypogonadal men with chronic inflammatory conditions reduce tumor necrosis factor-alpha (TNF-α), IL-1β, and IL-6, we suggest that testosterone therapy attenuates the inflammatory process and reduces the burden of disease by mechanisms inhibiting inflammatory cytokine expression and function. Mechanistically, androgens regulate the expression and function of inflammatory cytokines, including TNF-α, IL-1β, IL-6, and CRP (C-reactive protein). Here, we suggest that testosterone regulates multiple and overlapping cellular and molecular pathways involving a host of immune cells and biochemical factors that converge to contribute to attenuation of the inflammatory process.

## 1. Introduction

Considerable knowledge has been acquired regarding the cellular and molecular pathways of the inflammatory process [1,2]; however, much remains to be understood, including the role of sex steroid hormones in promoting or attenuating inflammatory responses. Herein, we attempt to review the contemporary literature regarding androgen modulation of the inflammatory response, and its implications for future research and therapeutic potential. 

In this review, we wish to focus our discussion on the effects of testosterone deficiency (i.e., hypogonadism) and testosterone therapy in men with inflammatory diseases, and the changes in the expression and function of inflammatory biomarkers, such as tumor necrosis factor-α (TNF-α), interleukin-1β (IL-1β), interleukin-6 (IL-6), and C-reactive protein (CRP). Our focus on these specific biological markers is, in part, due to recent advances in clinical applications of anti-inflammatory cytokines therapy for various clinical conditions [3,4,5]. For instance, adalimumab (Humira^®^), a TNF-α inhibitor, is the highest-selling drug today, and it is being used in various auto-immune diseases [6]. More recently, Ridker et al. [7,8] reported that IL-1β-blocking antibody prevented the recurrence of cardiovascular events, and reduced lung cancer incidence and mortality. Similarly, anti-IL-1β were also thought to ameliorate the symptoms of inflammatory bowel diseases, Crohn’s disease, and ulcerative colitis, and to attenuate the development of non-small cell lung cell (NSCLC) in smokers [9,10].

A host of pre-clinical and clinical studies demonstrate that testosterone reduces TNF-α, interleukin isoforms, and CRP, by various and often overlapping physiological mechanisms. Indeed, there are many other inflammatory markers, with various biological functions, that play a role in chronic inflammation and contribute to chronic diseases. However, for brevity’s sake, we will limit the scope of our discussion in this review to the aforementioned biomarkers. We begin by reviewing various inflammatory diseases in which the role of androgens has been explored; we then offer a larger biochemical overview of androgen-mediation within the inflammatory cascade.

## 2. Inflammatory Biomarkers Are Elevated in Men with Testosterone Deficiency and Reduced with Testosterone Therapy

Epidemiological and clinical studies reported increased levels of inflammatory biomarkers in response to androgen deficiency (Table 1) [11,12,13,14,15,16,17,18]. One common observation in these studies is that higher levels of IL-6, IL-1β, TNF-α, and CRP were noted in men with testosterone deficiency (hypogonadism). These findings are congruent with findings from pre-clinical studies in animal models (See the Biochemistry section below). Just as testosterone deficiency results in higher inflammatory marker expression, a number of studies demonstrated that testosterone therapy reduces the levels of such biomarkers [18,19,20,21,22,23,24,25,26] (Table 2). For example, Malkin et al. [19] demonstrated that testosterone treatment in hypogonadal men resulted in reductions in TNF-α and IL-1β, and in an increase in IL-10. Similarly, Kalinchenko et al. [23] reported that testosterone therapy for 30 weeks in 113 hypogonadal men resulted in reduction of IL-1β, TNF-α and CRP, but that IL-6 and IL-10 levels were unaffected. These observations are of clinical relevance, since therapeutic strategies of chronic inflammatory diseases utilize agents to reduce the expression and levels of inflammatory cytokines. Thus, testosterone therapy may be a potential tool in developing new therapeutic strategies.

## 3. Role of Androgens in Chronic Inflammatory Diseases

Reduced circulating testosterone levels have been associated with a host of autoimmune diseases concomitant with an increased production of inflammatory markers (CRP, TNF-α, IL-6) [14,27,28,29,30,31,32,33]. The anti-inflammatory properties of testosterone have been demonstrated in various models of diseases, including autoimmune encephalomyelitis, myasthenia gravis, diabetes, and coronary artery disease [34,35,36,37,38]. The possible role of testosterone in modulating inflammatory responses in various chronic inflammatory diseases (Table 3 and references therein) is discussed below.

### 3.1. Diabetes

Dhindsa et al. [39] demonstrated that the testosterone treatment of hypogonadal men with type 2 diabetes resulted in improvement in the expression of insulin-signaling genes (IR-β, IRS-1, AKT-2, and GLUT4) in adipose tissue; testosterone also significantly decreased circulating concentrations of free fatty acids, CRP, IL-1β, TNF-α, and leptin. Furthermore, Corrales et al. [20] showed that testosterone therapy in men with hypogonadism is associated with the reduction or complete abrogation of spontaneous ex vivo production of IL-1β, IL-6 and TNF-α by antigen-presenting cells (APCs).

### 3.2. Coronary Artery Disease

In patients with coronary artery disease, Wickramatilake et al. [47] showed that total testosterone levels were low, while hs-CRP levels were high as compared to control subjects. Low levels of total testosterone combined with high levels of hs-CRP and an abnormal lipid profiles appeared to be risk factors in atherogenesis. Indirectly, the positive correlation between testosterone and high-density lipoprotein (HDL) levels then reflected a protective role for testosterone in coronary artery disease (CAD). The inverse relationship between serum total testosterone and hs-CRP may indicate that low levels of endogenous testosterone contribute to low-grade chronic inflammation, thus promoting an atherogenic environment. Reports on the association between testosterone and hs-CRP are restricted to a few studies, but all of them show negative correlations (albeit with variable strength of relationships) [91,92].

### 3.3. Psoriasis

There are striking similarities between the molecular and inflammatory pathways in psoriasis and atherosclerosis [93]. Indeed, many studies have confirmed that patients with psoriasis have a significantly higher risk of being afflicted with obesity and diabetes mellitus [94]. Inflammation in psoriasis results in a Th1 lymphocyte cytokine milieu and an increased systemic inflammation [95,96,97,98]. In a series of 15 clinical cases, Saad et al. [25] reported that hypogonadal men with psoriasis treated with testosterone for a long period of time demonstrated significant benefits in reduced psoriasis area and reduced severity index (PASI), as well as physician global assessment for psoriasis (PGA) and reduced CRP levels. This preliminary pilot study suggests that testosterone therapy may be of potential benefit, and that it should be explored further.

### 3.4. Rheumatoid Arthritis

In multivariable analyses of almost 500,000 men, adjusted for the number of outpatient visits in the prior year and over 30 comorbid diseases, Baillargeon et al. [56] identified 123,460 men diagnosed with hypogonadism with no prior history of rheumatic autoimmune disease. Among these men, untreated hypogonadism was associated with an increased risk of developing any rheumatic autoimmune disease (HR = 1.33, 95% CI = 1.28, 1.38), rheumatoid arthritis (HR = 1.31, 95% CI = 1.22, 1.44), and lupus (HR = 1.58, 95% CI = 1.28, 1.94). These findings persisted with latency periods of one and two years. Patients diagnosed with hypogonadism who remained untreated with testosterone had an increased risk of developing rheumatic autoimmune disease, rheumatoid arthritis, and lupus.

Daily testosterone treatment for six months in men with rheumatoid arthritis (RA) [62,99] produced a significant increase in the number of CD8q T-cells, and a decrease in the CD4q (helper):CD8q T-cell ratio. The immunoglobulin M (IgM) rheumatoid factor concentration decreased significantly. A concurrent, significant reduction was noted in the number of affected joints, and in the daily intake of non-steroidal anti-inflammatory drugs. In another study in which testosterone treatment was administered to 57 postmenopausal women with RA, there was significant improvement in the pain score, ESR, and disability [100]. These findings suggest that testosterone deficiency may exacerbate RA, and that testosterone therapy appears to ameliorate symptoms. 

### 3.5. Crohn’s Disease

Crohn’s disease is an inflammatory chronic bowel disease that is characterized by an imbalanced production of pro-inflammatory mediators, and an increased recruitment of leukocytes to the site of inflammation. In a cumulative, prospective, registry study with an increasing number of men over time, Nasser et al. [26] reported on 92 hypogonadal men diagnosed with Crohn’s disease who received appropriate treatment for Crohn’s disease, and were also treated with testosterone. Another 14 hypogonadal men diagnosed with Crohn’s disease received treatment for Crohn’s disease, but did not receive testosterone. Both groups were followed for up to seven years. In men receiving testosterone, the Crohn’s Disease Activity Index declined from 239.36 ± 36.96 to 71.67 ± 3.26 at 84 months (*p* < 0.0001 vs. baseline), while C-reactive protein levels decreased from 12.89 ± 8.64 to 1.78 ± 1.37 mg/L at 84 months (*p* < 0.0001 vs. baseline). In addition, leukocyte count decreased from 11.93 ± 2.85 to 6.21 ± 1.01 × 109/L (*p* < 0.0001 at 84 months vs. baseline). The authors reported that no changes were observed in the untreated group of 14 men. This pilot study suggested that restoring testosterone to physiological levels in hypogonadal men with Crohn’s disease had a positive effect on the clinical disease course, with decrement in markers of inflammation.

### 3.6. Airway Diseases

It has been observed that the gender predominance of asthma shifts from males to females after puberty [101]. In fact, early menarche increases the risk of asthma occurrence [102], while the worsening of symptoms around menses is not an uncommon phenomenon [103]. Whether these epidemiologic findings are attributable to an excess of estrogen or progesterone, or a relative deficiency of testosterone, is unclear. Mileva et al. [104] reported their observations in 427 asthmatic men that low testosterone levels were more likely to be found in men with severe and moderate asthma compared with mild disease. In addition, low testosterone levels were also more likely to be found in patients with non-atopic than atopic disease [104]. It was also noted that asthmatic women with female fetuses are more likely to suffer asthma symptoms than women with male fetuses, postulating that a spillover of androgens may exert a protective effect on the mother [105].

Cellular and murine studies have elucidated mechanisms by which testosterone could exert protective effects against asthma seen at the population level. Mouse models show that males deprived of testosterone via castration will develop the same degree of airway inflammation as females when exposed to ovalbumin. Intact mice had less eosinophilic and lymphocytic infiltration, and lower splenic IL-4 expression than castrated males and female mice [106]. IL-4 expression is consistent with the Th2-type phenotypic response that is commonly seen in atopic asthma [107], and it is thought to be the prime driver for airways inflammation. In a separate experiment, male mice infected with Coxsackievirus in fact responded with a Th1 immunologic phenotype, while female mice demonstrated the Th2 phenotype. Administration of estradiol to the males equalized the ratio of IL-2/IFN-γ to IL-4-producing cells, suggesting a shift to the Th2 phenotype that is seen in female mice [108].

Likely, the expression of Th1 versus Th2 immunologic phenotypes is the result of a complex interplay between estrogens and androgens, but 5α-DHT and testosterone may suppress key triggers that are needed for IL-4 production, which is needed for progression to the Th2 response that is seen in allergic asthma. The exact points of suppression have not entirely been elucidated, but recent evidence suggests that 5α-DHT and testosterone suppress differentiation of Group 2 innate lymphoid cells (ILC2) in mouse and human blood models. These cells generate many of the necessary cytokines (including IL-4, as well as IL-13, IL-33, and others) that are needed for Th2 differentiation. ILC2 progenitors were found to be lower in female than male mice, and moreover, exposure of these progenitors to 5α-DHT in the presence of stimulatory cytokines blunted their maturation. ILC2 progenitors express androgen receptor (AR), and when exposed to the AR antagonist flutamide, there was an increase in ILC2 differentiation—killer cell lectin-like receptors G1 (KLRG1) (shown to suppress ILC2 function via E-cadherin) was reduced in these cells, suggesting that AR-signaling is needed for the active suppression of ILC2 maturation. Both orchiectomy and chimeric formation with AR-deficient bone marrow resulted in the elimination of the sex difference in ILC2 numbers and phenotype, but not ovariectomy)—again suggesting androgens are needed to maintain ILC2 homeostasis [66].

In a multi-faceted study, similar results were observed by Cephus and colleagues, [67] when ILC2 were isolated from male and female mice and exposed to different combinations of IL-33 (alone), IL-2 and -33, and IL-7 and -33. In two of the three, (IL-33 alone, and IL-2 and -33), there was less proliferation of the male ILC2 than female cells. When gonadectomized male mice were exposed to 5α-DHT, 17-β estradiol, progesterone, or a combination of estradiol and progesterone (or a control pellet), only the gonadectomized mice exposed to 5α-DHT had lower lung ILC2 numbers than the other groups. Androgens, therefore, not only seem to modulate the numbers of ILC2, but also their chemotaxis to lung tissue, as well as their cytokine expression profile (IL-5 and IL-13 expression was decreased in intact male mice and in gonadectomized mice exposed to 5α-DHT, compared to female mice and male mice receiving placebos; notably, exposure to female sex hormones had no effect on these cytokines compared to control). Though human ILC2 differential expression to sex hormones was not studied, Cephus and colleagues did quantify circulating ILC2 in men and women of reproductive age, finding increased numbers in asthmatics versus those without asthma, and higher levels in asthmatic women than men (healthy men and women had similar ILC2 levels). Taken together, the implication is that ILC2 may play a key role in the pathogenesis of airway inflammation, and testosterone and its derivatives could exert an important modulatory effect on this cell type [67].

Either independently or as a linked mechanism, testosterone and 5α-DHT may influence the production of leukotrienes via 5-lipoxygenase (5-LO) subcellular trafficking. Leukotrienes are important inflammatory mediators for both the innate and adaptive immune systems, and with regard to allergic asthma, they may specifically induce ILC2 activation and lung inflammation [109]. In neutrophils derived from female human blood, Pergola and colleagues observed that 5-LO mainly localized to the cytoplasmic compartment, whereas in male-derived neutrophils, 5-LO was distributed in both the cytoplasm and nucleus. Stimulation with lipopolysaccharide-containing compounds resulted in trafficking of the 5-LO from the cytoplasm to the nucleus in female-derived neutrophils, but not in male ones (where the distribution of 5-LO remained as it was in the un-stimulated state). Exposure of neutrophils from both genders to 5α-DHT (but not estradiol or progesterone) resulted in the redistribution of 5-LO from the cytosol to the nucleus; this effect was reversible. Treatment of neutrophils from females with 5α-DHT resulted in 5-LO product synthesis levels that were close to those seen in male-derived cells; in an interesting twist, 5-LO activity was similarly low in hyperandrogenic females than females with normal testosterone levels. Testosterone may exert its modulating effect through type 2 extracellular receptor kinase (ERK2) phosphorylation—a mechanism of testosterone regulation already mentioned above. Baseline ERK2 phosphorylation was higher in male than female-derived cells; administration of ERK inhibitors caused a female pattern of 5-LO distribution. 5α-DHT exposure caused the rapid phosphorylation of ERK2 in female neutrophils (female sex hormones did not have a similar effect), as did incubation of the female neutrophils with male cells [110]. Testosterone regulation of ERK phosphorylation and 5-LO trafficking (and thereby, 5-LO activity) may explain the sex differences seen in leukotriene-antagonist therapy (being more efficacious in pubescent females than males) [111].

The literature regarding the role of testosterone in chronic obstructive pulmonary disease (COPD) is sparser [78,112], but we call attention to one recent study performed by Baillargeon & colleagues [71], in which national-level data were reviewed for outcomes of COPD patients who were prescribed testosterone replacement therapy matched to non-users. Compared to non-users, middle-aged testosterone users had a 4.2% decrease in respiratory hospitalizations; older testosterone users had a 9.1% decrease. High rates of testosterone deficiency have been reported in COPD patients, perhaps due to systemic inflammation, glucocorticoid use, or chronic illness. Perhaps testosterone affected some of these factors directly, or it may have resulted in improved body composition, skeletal muscle strength and exercise capacity, resulting in the relief of symptoms typically leading to hospitalization among these patients. 

### 3.7. Multiple Sclerosis

Multiple sclerosis is a chronic inflammatory disease of the central nervous system with a pronounced neurodegenerative component. The role of androgens in the experimental autoimmune encephalomyelitis (EAE) model, which is the most commonly used model of multiple sclerosis (MS), has been investigated. Surgical castration of male animals caused a greater susceptibility to EAE and a greater severity of disease [113]. Testosterone treatment in castrated animals attenuated symptoms and reduced inflammation, concomitant with the decreased secretion of proinflammatory cytokines (TNF-α and IFN-γ) and the increased production of Th2 cytokines (IL-10) by CD4^+^ T lymphocytes [35,114]. Data from clinical trials on the effects of testosterone therapy in MS suggest improvements in this condition with testosterone administration [38,79,83]. In one study, [79] testosterone treatment in patients with MS significantly reduced delayed type hypersensitivity (DTH) recall responses and induced a shift in peripheral lymphocyte composition by decreasing CD4^+^ T-cell percentage and increasing natural killer (NK) cells. In addition, blood peripheral mononuclear cells (PBMC) production of IL-2 was significantly decreased, while TGFβ1 production was increased. Furthermore, PBMCs obtained during the treatment period produced significantly more brain-derived neurotrophic factor (BDNF) and platelet-derived growth factor (PDGF-BB). Increased production of BDNF and PDGF-BB suggests a potential neuroprotective effect by testosterone. It was shown that one year of treatment with testosterone was associated with an improvement of cognitive performance (measured by the PASAT test), and a slowing of brain atrophy [79].

### 3.8. Systemic Lupus Erythematosus (SLE)

A retrospective cohort study by Pakpoor et al. [84] estimated the rates for SLE following testicular hypofunction, after adjustments for a host of co-variables. The adjusted rate ratio (RR) of SLE following testicular hypofunction was 7.7 (95% confidence interval (95% CI) 2.5–18.1, *p* < 0.0001). The adjusted RR for testicular hypofunction following SLE was 6.5 (95% CI 2.1–15.1, *p* < 0.0001). The positive association between testicular hypofunction and SLE supports the hypothesis that low testosterone levels may influence the development of male SLE. The authors suggested that based on this large data set, an association between clinically diagnosed low levels of testosterone and SLE exist; however, this needs to be verified in future studies. Also, patients with hypergonadotropic hypogonadism are prone to the development of SLE. Recently, Chan et al. [115] reported on male-to-female transsexuals who developed SLE 20 years after sex-reassignment surgery and prolonged estrogen therapy. For extensive details on the role of androgens in modulation of the immune system in chronic inflammatory diseases, the readers are referred to a comprehensive review by Gubbels Bupp & Jorgensen [116].

## 4. Sex Steroid Hormones Modulate the Pathophysiology of Inflammation

**Figure 1:**
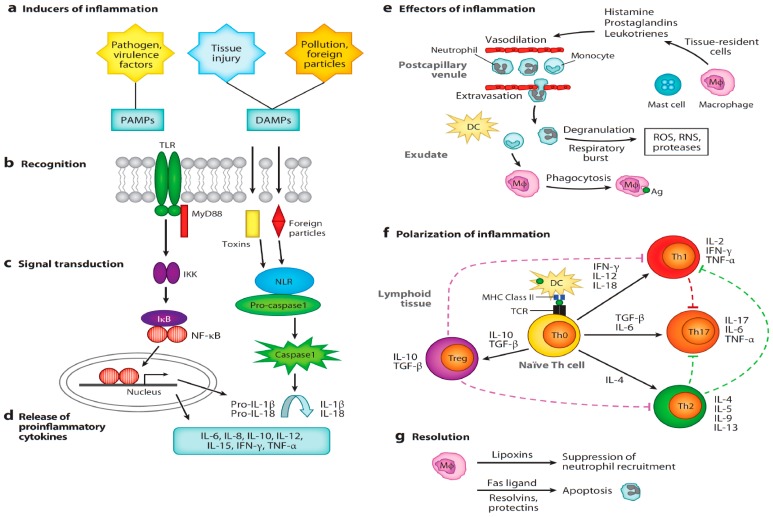
A primer of the inflammatory cascade. (**a**) Pathogens, tissue injury, and foreign particles induce inflammation. (**b**) Transmembrane TLRs and intracellular NLRs bind to PAMPs or DAMPs, respectively. (**c**) TLRs activate a MyD88-dependent signal transduction pathway that involves the phosphorylation of the inhibitory IκB protein by IKK. NF-κB is released from IκB, and it translocates to the nucleus where transcription is upregulated through binding to target inflammatory genes. NLRs signal the inflammasome, which activates caspase-1 to convert cytokines into active forms (IL-1β and IL-18), which then elicit inflammation after being released from the cell. (**d**) A variety of proinflammatory cytokines and chemokines are produced and released to promote effector functions of inflammation. (**e**) Blood-borne neutrophils and monocytes migrate to the site of disturbance by chemotaxis and selectively pass through endothelial cells to reach target sites (extravasation). This influx of cells is accompanied by protein-rich fluid, known as the exudate, and promotes edema (swelling). Mast cells and tissue-resident macrophages promote this migration by releasing histamine, leukotrienes, and prostaglandins, which have rapid effects upon the vasculature, including vasodilation and increased vascular permeability. Neutrophils release toxic compounds, including ROS, RNS, and various proteases, which are nonspecific and harm both the pathogen and host. Macrophages and dendritic cells participate in the phagocytosis of Ag. (**f**) These cells migrate to lymphoid tissue and prime naıve T-cells (Th0) to become polarized through the stimulation of the TCR by antigens bound to MHC class II receptors. Th0 cells differentiate into several different types of effectors and regulatory cells: Th1 cells (proinflammatory), Th2 cells (anti-inflammatory), Tregs (regulatory), and Th17 cells (proinflammatory). Depending upon the type of pathogen and other factors, the resulting Th population can be biased toward a proinflammatory, anti-inflammatory, or regulatory phenotype. Cytokines produced by polarized Th1 and Th2 are mutually inhibitory, whereas cytokines produced by Treg cells dampen both Th1 and Th2 responses. Th17 cells are highly proinflammatory and are regulated by the other Th subsets. Black-arrowed and dashed lines represent stimulatory and inhibitory actions, respectively. (**g**) Resolution of inflammation occurs when neutrophils promote the switch of leukotrienes produced by macrophages and other cells to lipoxins, which initiates the termination of inflammation. Fas ligand, resolvins, and protectins promote the apoptosis of neutrophils. Macrophages phagocytose apoptotic neutrophils and cellular debris. Abbreviations: Ag, antigen; DC, dendritic cell; DAMP, damage-associated molecular pattern; IκB, nuclear factor of kappa light polypeptide gene enhancer in B-cells inhibitor; IKK, inhibitor of kappa B kinase; IFN-γ, interferon-gamma; IL, interleukin; Mφ, macrophage; MHC, major histocompatibility complex; MyD88, myeloid differentiation primary response gene; NF-κB, nuclear factor kappa-light-chain-enhancer of activated B-cells; NLR, nucleotide binding domain and leucine-rich-repeat-containing receptors; PAMP, pathogen-associated molecular pattern; RNS, reactive nitrogen species; ROS, reactive oxygen species; TCR, T-cell receptor; TGF-β, transforming growth factor-beta; Th, T helper cell; TLR, Toll-like receptor; TNF-α, tumor necrosis factor-alpha; Treg, regulatory T-cell. (Noah T. Ashley, Zachary M. Weil, and Randy J. Nelson. Inflammation: Mechanisms, Costs, and Natural Variation Annu. Rev. Ecol. Evol. Syst. 2012. 43: 385–406; with permission from the publisher).

Inflammation is a multi-tiered pathophysiological process caused by perturbations of normal homeostasis, attributed to infection, injury or imbalances in biochemical modulators [1,2]. Triggered by the innate immune cell recognition of pathogen-associated molecular patterns (PAMPs) or damage-associated molecular patterns (DAMPs), soluble signaling molecules known as cytokines are released from affected cells into adjoining tissues and eventually the bloodstream, causing changes in vascular tone, permeability, and local blood flow, with the end-effect of recruiting further immune cells and proteins to the site(s) of injury. Most innate cell recognition is via transmembrane Toll-like receptors (TLRs), and intracellular nucleotide binding-domain receptors (NOD-like receptors) or nuclear leucine-rich repeat containing receptors (NLRs) and leucine-rich-repeat containing receptors, which (upon recognition of PAMPs and DAMPs) activate common signaling pathways that result in the promotion of NF-κB activity (nuclear factor kappa-light-chain-enhancer of activated B-cells (Figure 1) [117,118,119]. This results in the release of pro-inflammatory cytokines, such as IL-1β, IL-6, and TNF-α, among others, resulting in effector cell recruitment as well as the aforementioned physiologic changes (meant to enhance immune cell delivery to affected sites).

The contemporary literature suggests that testosterone elicits anti-inflammatory effects in various tissues and organs [19,21,33,120,121,122,123]. Considerable evidence exists suggesting that androgens and estrogens regulate the function of macrophages, monocytes, neutrophils, eosinophils, mast cells, and other cellular components of the immune system [124]. Neutrophils, mast cells, macrophages, B-cells, and T-cells all express the AR protein, suggesting a conserved role for androgens in cellular signaling and inflammation across the immune system [125,126,127,128].

### 4.1. General Overview of Androgen-Mediated Suppression of Inflammation

The relationship between androgen deficiency and increased inflammatory biomarkers has been investigated in several animal models. Freeman et al. [129] examined the effects of various testosterone doses in aged orchiectomized male rats on interleukin expression. Specifically, IL-2, IL-6, IL-10, IL-12, and IL-13 were elevated in animals with reduced testosterone levels; and decreased in animals treated with physiological levels of testosterone. More recently, Chen et al. [130] demonstrated that testosterone deficiency increased the expression of TNF-α and nitric oxide (NO) secretion in response to lipopolysaccharides (LPS) in rat splenocytes. Testosterone treatment of orchiectomized animals also significantly attenuated the LPS-elicited release of TNF-α and inducible nitric oxide (iNO) in a dose-dependent manner. It was suggested that testosterone may exert a protective role against inflammatory responses in the spleen.

LPS challenges of splenocytes revealed a marked elevation in the secretion of TNF-α [130]. When TNF-α secretion in intact and orchiectomized animals were compared after LPS administration, a marked increase in TNF-α release was noted in the orchiectomized group. Testosterone administration to orchiectomized animals reduced LPS-evoked TNF-α secretion. Orchidectomy promoted splenocyte proliferation rate in LPS-evoked splenocytes as compared with intact rats. However, testosterone treatment attenuated splenocyte proliferation in the castrated rats. These observations suggest that testosterone suppressed LPS-induced cell proliferation [130].

TNF-α is a critical factor in the pathogenesis of multiple inflammatory symptoms [131], and down-regulation of TNF-α reduces expressions of inflammatory factors in chronic inflammatory diseases [132,133]. Other studies in the rodent have similarly demonstrated that castration increases TNF-α secretion in the macrophage [134], and IL-6 in the serum and bone marrow [133,134,135,136]. The spleen is a unique and important innate as well as an adaptive immunity organ [137]. Emerging studies have suggested that down-regulation of p-ERK expression may contribute to anti-inflammatory effects [138,139,140].

Fijak et al. [141] investigated the effects of testosterone treatment in the inhibition of the development of experimental autoimmune orchitis (EAO) in a rat model. Testosterone treatment in an experimental model of EAO reduced EAO development by 17–33% as compared to 80% of animals developing disease in the EAO control group. Testosterone treatment prevented the accumulation of macrophages, and significantly reduced the number of CD4^+^ T-cells, with a strong concomitant increase in the number of regulatory T-cells (CD4^+^CD25^+^Foxp3^+^) compared with the EAO control. In addition, testosterone treatment reduced the expression of proinflammatory mediators such as MCP-1, TNF-α, and IL-6 in the testis, and secretion of Th1 cytokines such as IFN- and IL-2 by mononuclear cells isolated from testicular draining lymph nodes. These findings demonstrate that testosterone may exert a multifaceted protective effect during EAO development, likely via inhibition of Th1-specific cytokine production in testicular draining LNs. In the testis, T appears to induce an expansion of suppressive Tregs from naive T-cells, leading to an over-proportional representation of Tregs within the CD4^+^ T-cell population while simultaneously inhibiting the synthesis of proinflammatory mediators MCP-1, TNF-α, and IL-10.

Jin et al. [142], and Hong et al. [143], demonstrated that in in vitro studies, testosterone increased the protein and messenger RNA (mRNA) expression levels of tissue factor pathway inhibitor (TFPI), and inhibited NF-κB activity in human umbilical vein endothelial cells (HUVECs). Because NF-κB is a transcription factor that is known to be induced by cytokines and TNF-α, during inflammation, it is possible that testosterone inhibits TNF-α expression and attenuates inflammation. In addition, in human endothelial cells and mouse aorta, the androgen metabolite 3b-androstanediol (3-Adiol) was shown to be a potent inhibitor of TNF-α-mediated release of a series of adhesion molecules, cytokines, chemokines, and metallo-proteinases, as well as other molecules involved in the inflammatory response induced by TNF-α. This effect of 3b-Adiol also modulates the inflammatory response in macrophages, suggesting a common pathway in many cells [144,145]. D’Agostino et al. [146] demonstrated that AR is expressed in murine macrophage cell lines (J774), and that AR expression is reduced by testosterone treatment. More importantly, LPS treatment of J774 cell did not alter the expression of AR. In these cell lines, testosterone induced IL-10 synthesis. In cells treated with LPS, testosterone suppressed TNF-α expression and inducible NO production. These findings suggest that testosterone exerts anti-inflammatory effects by inhibiting TNF-α and NO, and increasing the synthesis of IL-10 [146]. Furthermore, the authors demonstrated that testosterone exerts anti-inflammatory effects and inhibits TNF-α, as well as nitric oxide, and that induces synthesis of IL-10. However, the mechanisms responsible for testosterone-induced cellular changes resulting in the decreased expression of specific cytokines and increased expression in others are not well understood. Yao et al. [147] showed that testosterone inhibited immune responses by reducing monocytes and the CD4^+^/CD8^+^ ratio, and inhibited the proliferative response of lymphocytes. In the proceeding sections, we will examine the potential mechanisms of the androgen regulation of expression of various cytokines, and their roles in the inflammatory responses. 

### 4.2. The Influence of Androgens on Specific Components of the Immune System

#### 4.2.1. Antigen Recognition

It has been suggested that testosterone deficiency may facilitate the activation of the TLR-4 pathway, and upregulate the expression of the TLR-4-regulated downstream target, ERK [130]. A significant increase in the protein levels of phosphorylated ERK (p-ERK) was demonstrated in the spleen of orchiectomized animals, which might lead to the production of inflammatory molecules. Testosterone treatment of orchiectomized animals reduced p-ERK expression levels in castrated rats as compared to the levels observed in intact rats. Rettew et al. [134] demonstrated that testosterone treatment in vitro of the RAW 264.7 mouse macrophage-like cell line produced significant decrease in the expression of the cell surface Toll-like receptor 4 (TLR4). Testosterone treatment decreased TLR4 expression only in cells derived in the absence of endogenous gonadal androgens. Cells isolated from sham operated animals or orchiectomized animals treated with testosterone failed to demonstrate such sensitivity. In addition, 5α-DHT treatment inhibited the expression of mRNA encoding TLR4 in human endothelial cells derived from neonatal tissue, and reduced LPS-mediated inflammatory mediator production by this cell type [144,145]. Further evidence that androgens down-regulate TLR4 was demonstrated in orchiectomized animals in which prostate TLR4 protein expression was elevated in castrated animals compared to intact animals [148].

#### 4.2.2. Neutrophils

Neutrophils are part of the innate system, and they are among the first responders to migrate to the injury site. Neutrophil activation stimulates the recruitment of monocytes from circulation into the inflamed tissues, in response to chemokines secreted from neutrophils and damaged tissues [149]. The infiltrated monocytes differentiate into macrophages, which in turn become the master regulators of the inflammatory response via the production of proinflammatory cytokines and growth factors to regulate immune responses and tissue regeneration [150,151]. Androgens are thought to play an important role in neutrophil development and function [128]. Aberrant inflammatory response from altered cytokine expression, adhesion molecules, or cell chemotaxis reduces neutrophil numbers. 5α-DHT has previously been reported to affect neutrophil-binding vascular adhesion molecule-1 (VCAM-1) in humans [124].

In a mouse model of AR knockout (ARKO), the neutrophil count was reduced by 90%, as compared to the neutrophil count in wild-type mice. Reductions in neutrophils were also reported in mouse models of testicular feminization (Tfm) [152] and subsequent to orchiectomy, albeit less dramatically than that observed in ARKO or Tfm mice models. Interestingly, restoring AR expression in the ARKO mouse model rescued granulocyte-macrophage progenitor neutrophil production, suggesting that AR may be critical for regulating neutrophil differentiation. Chuang et al. [152] proposed that androgens stimulate neutrophil production by enhancing the cellular signaling of granulocyte colony-stimulating factor (G-CSF).

#### 4.2.3. Monocytes and Adipocyte Chemoattraction

Chronic inflammation, obesity, and metabolic diseases are thought to be associated with age-related reduction in sex steroid hormones [153]. Adipocytes in obese individuals are thought to secrete monocyte chemoattractant protein-1 (MCP-1), a key chemokine that promotes the infiltration of monocytes/macrophages into adipose tissue, thus contributing to metabolic disorders. Monocytes play a central role in immunity, and they possess vast capacities to synthesize pro-inflammatory leukotrienes. In addition, monocytes are a major source of arachidonic acid metabolites, which play a physiological role in the inflammatory response [110,154,155,156]. The differentiation of monocytes into dendritic cells and macrophages further contributes to the immune response [110,155]. Morooka et al. [153] examined the effects of androgens on monocyte function, in particular the induction of MCP-1 expression in 3T3-L1 adipocytes co-cultured with RAW264.7 macrophages. Testosterone treatment suppressed TNF-α-induced MCP-1 and IL-6 expression in 3T3-L1 adipocytes via a mechanism that involves functional canonical Nuclear Factor-kappa B (NF-kB). These observations suggested that androgens down-regulate MCP-1 and IL-6 expression in 3T3-L1 adipocytes, and that androgen signaling regulates not only the infiltration of immune-cell types into adipose tissue, but also suppresses vicious feedback cycles that are associated with chronic inflammation in adipose tissue.

Separately, in an experimental model, it was shown that monocytes expressing AR and androgens modulate the signaling of inflammatory monocyte populations and monocyte chemotaxis, resulting in the attenuated expression of TNF-α [128]. Moreover, testosterone treatment independently inhibited the production of IL-6 by human peripheral blood monocytes [157,158,159], reduced lipopolysaccharide-stimulated prostaglandin E_2_ (PGE_2_) production, and regulated apoptosis in monocytic cell lines [27,62,99,110,155,159,160,161]. It should be pointed out that the exact molecular mechanisms by which androgens regulate monocyte function remains poorly understood. However, a few studies have suggested that the activation of extra-cellular receptor kinase (ERK) by androgens in monocytes repressed phospholipase D (PLD) and impaired 5-lipoxygenase (5-LO) activities. These observations suggest that, in monocytes, androgens are required for ERK activation, resulting in reduced PLD activity and diacylglycerol (DAG) levels. Other potential pathways by which androgens could modulate monocytes function in inflammation include the regulation of adaptor receptor proteins such as receptor for activated C kinase 1 (RACK1). Corsini et al. [162] demonstrated that in monocytes, activation of AR is critical to the action of androgens on (RACK1) expression and cytokine release. The activation of RACK 1 pathway is inhibited by flutamide, an AR antagonist. Also, inhibition of AR expression by small interfering RNA (siRNA), or inhibition of testosterone conversion to 5α-DHT by finasteride also attenuated RACK1 function, suggesting a critical role for androgen in this pathway. Corrales et al. [123,163], also investigated the effects of testosterone therapy on the number and the functionality of peripheral blood (classical and non-classical) monocytes and dendritic cells (myeloid and plasmacytoid) from men receiving testosterone therapy, as determined by the expression of CD107b (lysosome-associated membrane protein 2 (LAMP-2)). Testosterone treatment induced the overexpression of CD107b in circulating monocytes and dendritic cells derived from men with testosterone deficiency.

#### 4.2.4. Macrophages

Macrophages (as well as neutrophils and mast cells, discussed separately) express steroid hormone receptors [124]. The expression of TNF-α, IL-6 and IL-1β is reduced in rodent macrophage cell models and in human monocytes obtained from younger individuals [146,158,159,164,165]. Treatment with physiological or pharmacological levels of testosterone significantly reduced TNF-α expression and secretion in human monocyte-derived macrophages (HMDMs), and reduced IL-1β expression (though IL-6 expression was not affected) [164]. In a rat model of experimental autoimmune orchitis, androgen administration reduced disease severity, down-regulated TNF-α, IL-6 and MCP-1 mRNA expression, and inhibited macrophage recruitment (with a concomitant increase in the number of regulatory T-cells). Androgens have been shown to enhance proinflammatory cytokine production by macrophages in wound healing, but they also inhibit cytokine production after traumatic-hemorrhagic shock and burns [128]. It is possible that testosterone is necessary for maintaining overall inflammatory homeostasis.

In vitro studies showed that testosterone treatment diminished the production of TNF-α, IL-1β, and IL-6 in human macrophages and human monocytes. Furthermore, testosterone treatment stimulated the expression of IL-10 [19]. Corcoran et al. [164] proposed that testosterone modifies the inflammatory response by attenuating cytokine expression in human macrophages. In cultured human monocyte-derived macrophages (HMDMs) obtained from healthy men and treated with physiological and supra-physiological testosterone concentrations and stimulated with oxidized low-density lipoproteins, the expression of TNF-α and IL-1β were reduced, but testosterone treatment did not affect IL-6, or CRP expression levels in this experimental system. These findings suggest that androgens exert anti-inflammatory effects by reducing macrophage TNF-α and IL-1β expression. These observations are consistent with the findings of Lai et al. [128], who suggested that AR modulates TNF-α expression in macrophages. In addition, D’Agostino et al. [146] showed that testosterone treatment of macrophage cell line J774 exerted an anti-inflammatory effect by inhibiting TNF-α and inducible nitric oxide (iNO) levels, and induced IL-10. In another study by Friedl et al. [166] in which the authors investigated the effect of increasing doses of testosterone (0.1–40 nM) treatment on iNO synthesis in murine macrophages (RAW 264.7 cells) stimulated by LPS, a dose-dependent inhibition of iNO synthesis was reported. Since the iNO pathway is involved in immune defenses, testosterone-induced iNOS inhibition may represent another mechanism by which macrophages modulate the inflammatory pathway.

Another potential mechanism of androgen action in macrophages may be related to regulating Toll-like receptor 4 (discussed in other contexts above). Rettew et al. [134] investigated the acute effects of testosterone on the expression of TLR4, in isolated mouse macrophages. The testosterone treatment of macrophages in vitro elicited a significant decrease in TLR4 expression, and sensitivity to a TLR4-specific ligand. Furthermore, castrated mice were significantly more susceptible to endotoxic shock, and macrophages isolated from castrated animals had significantly higher levels of TLR4 cell surface expression than those derived from sham-operated (intact) mice. More importantly, these effects were not apparent in castrated animals treated with exogenous testosterone. As such, these data represent important pleotropic mechanisms underlying the immunosuppressive effects of androgens.

#### 4.2.5. Mast Cells

Mast cell activation and their signaling are very complex, and they take part in acute responses, including leukocyte infiltration and initiation of the acquired immunity. Their spectrum of activities encompasses the chronic phase of persistent inflammation, tissue remodeling, and fibrosis [167]. Men with increased prostatic inflammatory activity exhibit reduced levels of 5α-DHT, suggesting a possible role for androgens in the migration of inflammatory cells. In experimental animal model studies, animals that were treated with 5α-DHT, showed higher cellularity, highlighting mast cells and fibroblasts. Cellularity and neovascularization are processes of the inflammatory phase, and testosterone treatment reduced the number of activated mast cells in prostatic epithelium in the castrated animal model [168]. Androgens were also reported to modulate the function of human skin mast cells (MCs) by reducing IL-6 production [169]. The susceptibility of IL-6 regulation by androgens appears to be mediated by NF-kB related mechanism [170,171].

#### 4.2.6. Humoral Immunity

**Figure 2:**
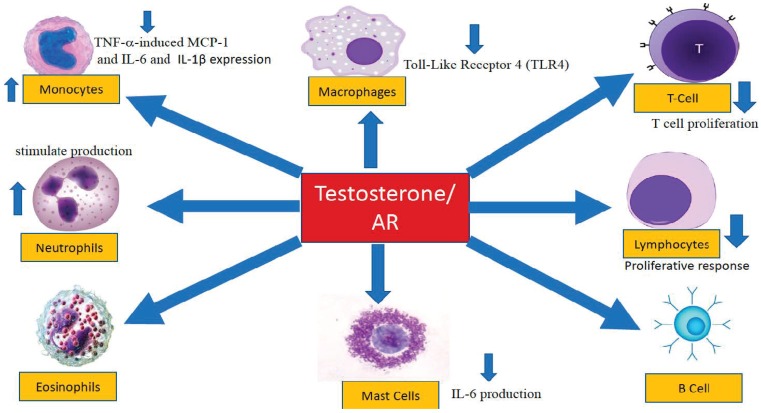
Testosterone modulates the innate and adaptive immune compartments, including monocytes, macrophages, neutrophils, eosinophils, mast cells, lymphocytes, T-cells, and B-cells.

T- and B-lymphocytes are central players in the adaptive response. Activation of these cells requires multiple pathways, some of which are regulated by androgen signaling. In animal models, the role of AR in differentiation and activation of T-cells and B-cells was thought to be critical. In T-lymphocytes, androgens suppress T-cell proliferation and modulate the balance of Th1 and Th2 responses. In contrast, androgens negatively regulate B-lymphocytes [128]. Androgens regulate the number and function of circulating lymphocytes and monocytes (Figure 2). Yao et al. [147] showed that while the absolute and relative numbers of monocytes decreased, the lymphocyte subpopulations determined by flow cytometry indicated an increase in CD8^+^ T-cells, whereas the CD3^+^, CD4^+^ and CD8^+^ T-cells remained unchanged. It was concluded that the immunosuppressive effects of testosterone may be attributed to a decline in number of monocytes, CD4^+^/CD8+ ratio, and proliferative activities, together with an increase of CD8^+^ T-cells in the animal model.

Testosterone inhibits the immune responses by reducing the monocytes and the CD4^+^/CD8^+^ ratio, and inhibiting the proliferative response of lymphocytes. Testosterone treatment inhibits production of IL-6 by human peripheral blood monocytes. Blockade of the AR expression by siRNA, or inhibition of testosterone conversion to 5α-DHT by finasteride attenuated RACK1 function, suggesting that androgens exert a critical role for in this pathway. Testosterone reduces the expression of proinflammatory mediators such as MCP-1, TNF-α and IL-6, and secretion of Th1 cytokines such as IFN- and IL-2 by mononuclear cells. Testosterone blocks T-cell activation and Th1 differentiation. In addition, testosterone inhibits thymic cellularity, and bone marrow B-cells and B-cell precursors and auto-antibodies. In sepsis, testosterone increases neutrophil counts and chemokines and pro-inflammatory cytokines. Testosterone stimulate neutrophil production by enhancing the cellular signaling of granulocyte colony-stimulating factor (G-CSF). Testosterone reduces TNF-α expression and secretion in human monocyte-derived macrophages (HMDMs), and reduces IL-1β expression. Testosterone prevents the accumulation of macrophages, and significantly reduces the number of CD4^+^ T-cells, with a strong concomitant increase in the number of regulatory T-cells (CD4^+^CD25^+^Foxp3^+^). Testosterone treatment in mouse macrophage-like cell lines produced significant decrease in the expression of cell surface TLR4. Testosterone reduces IL-6 production in human skin MCs. In T-lymphocytes, androgens suppress T-cell proliferation and modulate the balance of Th1 and Th2 responses. In contrast, androgens negatively regulate B lymphocytes. Testosterone also suppresses TNF-α-induced MCP-1 and IL-6 expression in 3T3-L1 adipocytes via a mechanism that involves functional canonical NF-kB.

Androgens may also impact humoral immunity via other mechanisms. As proposed in Figure 2, androgens attenuate T-cell activation and Th1 cell differentiation in the periphery, as well as modulating B-cell precursors and auto-antibody production. Androgens elicit inhibitory effects on T- and B-cell development, and dampen T-cell activation and inhibit Th1 differentiation in the periphery [172]. All these biochemical processes are believed to be modulated via the androgen receptor (AR) signaling pathways involving various immuno-cellular components.

Testosterone treatment produced a shift from Th1 to Th2 response by reduction in TNF-α or IL-6 secretion, inhibition of proliferation of T-cells, and T-cell apoptosis in experimentation in vitro [36,114,146,173,174,175]. Marked decreases in IL-2, IL-6, IL-10, IL-12, and IL-13 occur in animals deprived of testosterone, and these cytokines are restored by testosterone treatment [129]. AR activity reduces the proliferation of CD4^+^ T-clones, and suppresses the inflammatory response of human nonprofessional antigen-presenting cell cultures to inflammatory stimuli, suggesting that testosterone plays an important role in the anti-inflammatory function of T-cells [35].

## 5. Discussion

Considerable contemporary literature exists examining the effects of sex steroid hormones on the pathophysiology of inflammation [27,59,60,62,63,99,124,161]; however, no universal mechanism (s) has been advanced to explain such complex and interwoven pathways between androgen action and the attenuation of the inflammatory processes. The benefits of testosterone therapy in ameliorating or attenuating the symptoms of several chronic inflammatory diseases have been reported (Table 3). An example of the benefits of testosterone therapy are the improvements noted in patients with Crohn’s disease. Crohn’s disease patients received standard treatment (without testosterone), apparently without benefits other than maintaining the disease under control during active flare up phases. However, testosterone therapy not only improved autoimmune disease symptoms in this condition, but also provided added benefits such as improved sexual function, improved body composition, etc. An increase in muscle mass may be the most important benefit in debilitating diseases such as Crohn’s and COPD. We should point out that many auto-immune diseases are difficult to treat and cannot be cured, but their symptoms can be clinically managed. Many of the available treatment modalities provide temporary amelioration of symptoms. For this reason, testosterone therapy in diseases such as Crohn’s disease and psoriasis is of considerable clinical relevance. 

An anti-inflammatory effect of androgens has been reported in a number of studies [176,177,178]. One key observation is that raising serum testosterone concentrations to physiological levels in hypogonadal patients, by treatment with parenteral testosterone, along with improving metabolic profile, significantly reduces liver transaminases, CRP, and circulating TNF-α levels [23,24,179]. Because it has been demonstrated that increased levels of TNF-α are mechanistically linked to endothelial injury in different vascular beds and they directly suppress endothelial NOS expression, it is not surprising that inflammation with increased levels of TNF-α contributes to cardiovascular disease. Several recent studies in the animal model by Vignozzi and colleagues [180,181,182,183,184,185,186] reported that testosterone treatment in rabbits fed a high fat diet significantly ameliorated lipid accumulation and liver inflammation, and decreased liver TNF-α expression and circulating TNF-α plasma levels [184]. Therefore, testosterone therapy of hypogonadal men may blunt liver inflammation, thus counteracting the development of metabolic syndrome and associated cardiovascular disease.

Anti-TNF therapy is the mainstay for the treatment of moderate-to-severe inflammatory bowel disease (IBD), including Crohn’s disease (CD) and ulcerative colitis (UC) [3]. Recent clinical trials with interleukin-6 receptor monoclonal antibody tocilizumab (TCZ) and tumor necrosis factor inhibitors (TNFi) for the treatment of rheumatoid arthritis demonstrated improvements with anti-TNF-α therapy and anti-IL-6 receptor therapy [4]. Also, vedolizumab therapy was shown to improve ulcerative colitis and Crohn’s disease [5]. Since testosterone deficiency increases the levels of inflammatory cytokines (Table 1), and testosterone therapy in hypogonadal men with chronic inflammatory conditions also reduce TNF-α and IL6 (Table 2), it is possible that testosterone therapy reduces the levels and activities of these inflammatory cytokines, and therefore reducing the burden of disease, and the amelioration of symptoms in such clinical conditions (Table 3).

On the cellular and molecular level, androgens, via interactions with the AR, have been demonstrated to play an important role in regulating several cellular functions in the immune system (Figure 2) [19,124,127,128,130,134,144,145,146,147,148,152,153,154,155,156,157,158,159,161,162,164,165,166,170,172]. Data derived from animal model studies, as well as epidemiological and clinical studies (Table 1 and Table 2) strongly suggest that androgens regulate the expression and function of inflammatory cytokines, including TNF-α, IL-1β, IL-6, and CRP, among others. The findings from studies on testosterone therapy in men with testosterone deficiency have suggested that testosterone therapy attenuates the expression and function of a diverse number of inflammatory cytokines (Table 2). Therefore, it was postulated that androgens attenuate inflammatory responses, as shown in a host of chronic diseases such as Crohn’s disease psoriasis, rheumatoid arthritis and allergic asthma (Table 3) [28,29,30,31,93,94,95,96,97,98,100,101,102,103,104,105,106,107]. Because inflammation is a complex pathophysiological process, it is important to point out that androgens regulate multiple and overlapping molecular pathways involving a host of immune cells and biochemical factors that converge to contribute to the attenuation of the inflammatory process. Clearly, more fundamental basic science and clinical research studies are needed to accurately delineate such pathways and shed more light on the molecular mechanism of androgen action in regulating these complex processes. It is important to emphasize that a host of case studies, observational studies, and clinical trials presented data to suggest that androgen deficiency exacerbates inflammation (Table 1), and that testosterone therapy attenuates inflammation (Table 2 and Table 3). Such findings should pave the way for well-thought out and well-designed studies to examine this very important clinical issue, especially in elderly men with testosterone deficiency.

## Figures and Tables

**Table 1:** Effects of testosterone deficiency (hypogonadism) on the expression and/or function of inflammatory markers.

Study	Study Design	Major Findings	Authors Comments
Maggio et al., 2006 [11]	This study included 497 male participants, aged 65 years and older, excluded 22 men taking glucocorticoids, exogenous androgens, or antibiotic treatment and eight who had been recently hospitalized. The final analysis included 467 men (mean age, 74.8 ± 7.0 years; range, 65–97 years).	Older men tended to have higher levels of IL-6, TNF-α, IL-1β, and C-reactive protein (CRP), but the levels of soluble IL-6 receptor (sIL-6r) were not different. Levels of sIL-6r were inversely related to total testosterone and bioavailable testosterone. IL-6 was not associated with total testosterone or bioavailable testosterone. After adjusting for age and multiple confounders, the negative correlation between sIL-6r and testosterone and bioavailable testosterone was maintained. No independent association of testosterone or bio-availability with TNF-α or IL-1β CRP was found.	Testosterone was significantly, inversely and independently associated with sIL-6r, but not with IL-6, IL-1β, TNF-α, or CRP. Study findings suggest that testosterone may decrease inflammation by reducing the production of sIL-6r.
Kupelian et al., 2010 [12]	Cross-sectional observational survey of a random sample of 2301 racially and ethnically diverse men aged 30–79 years. Analyses were conducted on 1559 men, with complete data on CRP and sex hormone levels.	About 40% of men were overweight with body mass index (BMI) of 25.0–29.9 and one-third were obese (BMI ≥ 30). Over half of the analysis samples reported the use of anti-inflammatories or other medications that could affect CRP levels. One-third of men were at moderate cardiovascular risk (CRP levels 9.5–28.5 nmol/L) while almost 20% were at high risk (CRP >28.5 nmol/L). Strong correlation between testosterone/SHBG (sex hormone binding globulin) and CRP.	A robust inverse correlation of testosterone and sex hormone-binding globulin with CRP levels. These findings provide evidence supporting the hypothesis that modulation of inflammatory processes is a potential pathway by which androgens could affect cardiometabolic risk and associated conditions such as metabolic syndrome, diabetes, and cardiovascular disease.
Bobjer et al., 2012 [13]	Sub-fertile hypogonadal men (*n* = 20) and eugonadal men (*n* = 20) (mean age 37 years, standard deviation (SD) = 4.3 and age-matched controls (*n* = 20) were assessed for the levels of inflammatory markers.	Macrophage inflammatory proteins a and 1b (MIP1a & MIP1b and TNF-α all showed negative associations with testosterone levels, while MIP1a and TNF-α also showed negative association with calculated free testosterone (cFT) levels. Compared with men exhibiting normal testosterone and cFT levels, TNF-α levels were higher in men with subnormal levels of testosterone and cFT. Also, MIP1 levels were higher in men with subnormal levels of testosterone.	Higher levels of proinflammatory biomarkers were noted in young men with reduced testosterone, even in the absence of concurrent metabolic disease. These findings suggest that testosterone levels are directly associated with low grade systemic inflammation, which may contribute to the development of several adverse health effects previously associated with androgen deficiency. It appears that TNF-α and MIP1 are strongly associated with low testosterone in men not suffering from serious systemic disease.
Tsilidis et al., 2013 [14]	Data from 809 adult men in the National Health and Nutrition Examination Survey 1999–2004 were analyzed by geometric means and 95% confidence intervals for CRP and white blood cell (WBC) concentrations by sex steroid hormones and sex hormone binding globulin (SHBG), using weighted linear regression models.	Total and calculated free estradiol (E_2_) were positively associated with both CRP and WBC concentrations. SHBG concentrations were inversely associated with WBC count, but not with CRP. These cross-sectional findings are consistent with the hypothesis that higher androgen and lower estrogen concentrations may have an anti-inflammatory effect in men.	Testosterone and cFT are modestly inversely associated with CRP concentrations, and that total and calculated free estradiol are modestly positively associated with CRP and WBC.
Burney et al., 2012 [15]	Patients with cancer cachexia (*n* = 45) and cancer without cachexia (*n* = 50), as well as non-cancer controls (*n* = 45). Total testosterone, bioavailable testosterone, CRP, and IL-6 were measured in plasma. Functional performance was assessed by the Eastern Cooperative Oncology Group and Karnofsky Performance Scales, and sexual function was assessed by the International Index of Erectile Function (IIEF).	Low testosterone-levels were noted in more than 70% of cancer cachexia cases. Total testosterone was lower in cancer cachexia compared to cancer without cachexia. Cancer cachexia patients had lower bioavailable testosterone (BAT) and grip strength, IIEF scores, appendicular lean body mass, and fat mass, and higher IL-6 and CRP compared to controls.	Patients with cancer cachexia have lower grip strength, testosterone, fat mass, and a lean body mass with higher bone resorption, high sensitivity-CRP (hs-CRP), and IL-6, and poor functional status and erectile function, suggesting that cancer cachexia patients exhibited higher degrees of inflammation.
Tremellen et al., 2017 [16,17]	Men (*n* = 50) aged between 21 and 50 years (mean 35.1 ± 6.8 years) recruited from a private fertility clinic. BMI, waist circumference and % body fat, inflammatory status (serum CRP, IL-1β, IL-6, TNF-α, and lipopolysaccharide-binding protein (LBP)) and testicular endocrine function (serum testosterone, estradiol, anti-Mullerian hormone, luteinizing hormone (LH) and follicle stimulating hormone (FSH) were measured.	The mean (± SD) age, BMI, percentage body fat, and waist circumference of participants was 35.1 ± 6.8 years, 26.96 ± 3.5 kg/m2, 23.6 ± 6%, and 93.2 ± 9.5 cm, respectively. Inflammatory status and all three measures of adiposity, were positively correlated with serum IL-6 and CRP, but not with IL-1β or TNF-α. CRP was an overall marker of inflammation, and a positive correlation was observed between both CRP and the cytokines IL1β and IL-6, but not with TNF-α, CRP was negatively correlated with total testosterone (*r* = −0.471, *p* = 0.001,) but not calculated free testosterone (*r* = −0.238, *p* = 0.11). A significant negative relationship between serum IL-6 and testosterone (*r* = −0.516, *p* < 0.001), was observed.	Male adiposity was associated with both metabolic endotoxemia and an increase in serum IL-6, with this heightened inflammatory response being associated with a decline in both Leydig (testosterone) and Sertoli cell (anti-Mullerian hormone) function.

**Table 2:** Effects of testosterone therapy on inflammatory biomarkers.

Study	Type of Study	Main Findings	Comments
Ng et al., 2002 [18]	Three-month, randomized, double-blind, placebo-controlled clinical trials. In one group, 18 men were assigned to 5α-DHT, and 19 men were assigned to a placebo. In a second group, 20 men were assigned to recombinant human chorionic gonadotropin (rhCG), and 20 men were assigned to a placebo.	Median CRP levels were 1.65 and 1.50 mg/L, mean sICAM-1 levels were 228 and 224 ng/mL, and mean soluble vascular adhesion molecule-1 (sVCAM-1) levels were 861 and 895 ng/mL in placebo- and 5α-DHT-assigned treatment groups, respectively. rhCG had no significant effect on either CRP, sVCAM-1, or sICAM-1 compared with the placebo.	5 α-DHT replacement in older men for three months had no significant effects on the serum levels of CRP, sICAM-1, or sVCAM-1. rhCG administration had no effect on serum inflammatory markers in older men, suggesting that the conversion of testosterone to estrogens may not have a significant effect on circulating ICAM-1, VCAM-1, or CRP in older men.
Malkin et al., 2004 [19]	A randomized, single-blind, placebo-controlled, crossover trial of testosterone therapy in hypogonadal men.	Testosterone therapy reduced serum cytokines TNF-α and IL-1β, and an increase in IL-10. The reduction in IL-1β only approached significance. The reduction of TNF-α and IL-1β was positively correlated.	This study suggests that testosterone therapy reduces inflammatory cytokine levels.
Corrales et al., 2006 [20]	Thirteen men >55 years with type-2 diabetes were enrolled in the study. Eight healthy men with neither diabetes mellitus, nor any of the components of the metabolic syndrome, of similar age, were studied in parallel as a control group. Analyses were performed at baseline and 1, 3, 6 and 12 months after treatment with 150 mg testosterone enanthate every two weeks in the 13 men with type-2 diabetes.	After testosterone therapy, the number of peripheral blood monocytes capable of spontaneously producing IL-1β, IL-6, and TNF-α became undetectable, both at the first follow-up time point and at the end of therapy. Also, the percentage of CD33hi myeloid dendritic cells (DCs), and that of plasmacytoid DCs capable of spontaneously producing IL-6 and TNF-α also became undetectable. Testosterone therapy was associated with a decreased number of TNF-α secreting plasmacytoid DCs at month 12.	It was suggested that testosterone therapy in men beneficially alters cytokine balance and may reduce inflammation.
Kapoor et al., 2007 [21]	Double-blind placebo-controlled crossover study of testosterone therapy in 20 hypogonadal men with type 2 diabetes and >30 years.	There was a significant inverse correlation between baseline IL-6, CRP, and total testosterone.	Baseline testosterone levels correlated inversely with IL-6 and CRP, suggesting that testosterone may regulate the expression of inflammatory cytokines.
Nakhai-Pour et al., 2007 [22]	A randomized, double-blind, placebo-controlled trial of 237 men with serum testosterone levels <13.7 nmol/L and aged 60 to 80 years were treated with testosterone undecanoate (TU) or placebo for 26 weeks.	Median hs-CRP was 2.20 vs. 2.00 mg/L in the testosterone and the placebo group, respectively. Neither baseline testosterone level, nor age, or baseline CRP level modified the effect of testosterone supplementation on CRP levels.	This study suggested that 26 weeks of T therapy had no effect on serum hs-CRP levels in elderly men.
Kalinchenko et al., 2010 [23]	This was a double-blinded, placebo-controlled phase III trial of 170 men aged 35–70. Testosterone therapy was followed up for 30 weeks (*n* = 113; or placebo, *n* = 71). One hundred- and five-men receiving testosterone and 65 receiving a placebo completed the trial.	Testosterone therapy reduced the levels of TNF-α and CRP significantly, but not those of IL-1β. Levels of IL-6 and IL-10 were not affected by testosterone. Changes in CRP were correlated with changes in total and free testosterone.	This study suggested that testosterone therapy reduced levels of markers of inflammation.
Traish et al., 2014 [24]	Long-term observational registry study, throughout a 5-year period.	Cumulative registry study of 255 men, aged between 33 and 69 years (mean 58.02 ± 6.30) with subnormal plasma total T levels (mean: 9.93 ± 1.38; range: 5.89–12.13 nmol/L), as well as at least mild symptoms of TD assessed by the Aging Males’ Symptoms Scale. Testosterone therapy was followed up to 60 months.	Long-term testosterone therapy markedly reduced the levels of CRP in hypogonadal men.
Saad et al., 2015 [25]	In a single-center, cumulative, prospective registry study, hypogonadal men with psoriasis were investigated.	Men (*n* = 15) with a previous diagnosis of psoriasis received testosterone therapy for up to 93 months. Scores on the Psoriasis Area and Severity Index and Physician Global Assessment for Psoriasis showed significant improvement for the first 24 months. These improvements were sustained with continued testosterone therapy.	Levels of CRP, a biochemical indicator of inflammation, declined significantly.
Nasser et al., 2015 [26]	This study was a cumulative, prospective, registry with an increasing number of men with Crohn’s disease receiving testosterone over time. In total, 92 men received parenteral testosterone undecanoate 1000 mg/12 weeks for up to 7 years. Fourteen men opted not to receive testosterone and served as a comparison group.	In men receiving testosterone, the Crohn’s Disease Activity Index declined from 239.36 ± 36.96 to 71.67 ± 3.26 at 84 months (*p* < 0.0001 vs. baseline). C-reactive protein levels decreased from 12.89 ± 8.64 to 1.78 ± 1.37 mg/L at 84 months (*p* < 0.0001 vs. baseline). Leukocyte count decreased from 11.93 ± 2.85 to 6.21 ± 1.01 × 109/L (*p* < 0.0001 at 84 months vs. baseline).	The authors suggested that normalizing serum testosterone in hypogonadal men with Crohn’s disease had a positive effect on the clinical course, also evidenced by biochemical parameters.

**Table 3:** Possible Benefits of Testosterone in Various Inflammatory Conditions.

Chronic Inflammatory Disease	Reported Studies	General Comments from Various Studies
Type 2 Diabetes Mellitus	Dhindsa et al., 2016 [39] Hackett et al., 2018 [40] Hackett et al., 2018 [41] Shigehara et al., 2018 [42] Fink et al., 2018 [43] Groti et al., 2018 [44] Saad, 2017 [45] Haider et al., 2017 [46]	Testosterone treatment in hypogonadal diabetic men resulted in a significant fall in circulating concentrations of free fatty acids, C-reactive protein, interleukin-1β, tumor necrosis factor-α, and leptin (*p* < 0.05), concomitant with weight loss and an improvement in glycemic control, endothelial function, sexual function, and reduced mortality.
Coronary Artery and Vascular Diseases	Wickramatilake et al., 2014 [47] Kloner et al., 2016 [48] Pongkan et al., 2015 [49] Etminan et al., 2015 [50] Nettleship et al., 2009 [51] Traish et al., 2017 [52] Cheetham et al., 2017 [53] Anderson et al., 2016 [54] Sharma et al., 2015 [55]	An association between low testosterone levels and the presence of atherosclerosis, coronary artery disease, and coronary events was noted in a number of studies. Testosterone concentrations were lower, and highly-sensitive C-reactive protein levels were higher in coronary artery disease patients as compared with controls, and testosterone therapy may have a potential as a therapeutic agent in treating heart failure, angina, and myocardial ischemia, and testosterone therapy may exert cardioprotective effects.
Psoriasis	Saad et al., 2016 [25]	Testosterone therapy in hypogonadal men with psoriasis produced considerable improvements in scores of the Psoriasis Area and Severity Index and Physician Global Assessment for Psoriasis in the first 24 months, and these improvements were sustained thereafter. C-reactive protein levels declined significantly.
Rheumatoid Arthritis	Baillargeon et al., 2016 [56] Fisk et al., 1950 [57] Ganesan et al., 2012 [58] Cutolo et al., 2009 [59] Cutolo et al., 2006 [60] Pope et al., 2002 [61] Cutolo et al., 2002 [62] Cutolo et al., 2000 [63] Hall et al., 1996 [64] Wimer et al., 1973 [65]	Serum testosterone levels are inversely correlated with rheumatoid arthritis (RA) activity. Testosterone is significantly reduced in inflamed synovial tissue/fluids during active disease as a consequence of the inflammatory reaction, which supports a pro-inflammatory milieu in RA joints. Patients diagnosed with hypogonadism who were not treated with testosterone had an increased risk of developing any rheumatic autoimmune disease and RA, and lupus and testosterone therapy produced improvements in patients’ symptoms.
Crohn’s Disease	Nasser et al., 2015 [26]	Low circulating levels of testosterone are common in males with chronic obstructive pulmonary disease. It is possible that low anabolic hormones will reduce muscle mass and eventually result in a diminished muscle function. Normalizing serum testosterone in hypogonadal men with Crohn’s disease had a positive effect on the clinical course, also evidenced by biochemical parameters.
Airway Diseases	Laffont et al., 2017 [66] Cephus et al., 2017 [67] Montaño et al., 2014 [68] Canguven & Albayrak, 2011 [69] Kamischke et al., 1998 [70] Baillargeon et al., 2018 [71] Atlantis et al., 2013 [72] Samaras et al., 2012 [73] Velema et al., 2012 [74] Svartberg, 2010 [75] Puhan & Schünemann, 2005 [76] Casaburi et al., 2004 [77] Creutzberg & Casaburi, 2003 [78]	Testosterone therapy may slow disease progression in patients with chronic obstructive pulmonary disease (COPD). Androgens limits IL-33-driven lung inflammation through a cell-intrinsic inhibition of ILC2 expansion. In vivo, testosterone attenuated Alternaria-extract-induced IL-5+ and IL-13+ ILC2 numbers and lung eosinophils by intrinsically decreasing lung ILC2 numbers, as well as by decreasing expression of IL-33 and thymic stromal lymphopoietin (TSLP), ILC2-stimulating cytokines. Collectively, these findings provide a foundational understanding of sexual dimorphism in ILC2 function.
Multiple Sclerosis	Gold et al., 2008 [79] Gold & Voskuhl., 2009 [38] Gold & Voskuhl, 2006 [80] Collongues et al., 2018 [81] Ziehn et al., 2012 [82] Sicotte et al., 2007 [83] Dalal et al., 1997 [35]	Testosterone demonstrated an anti-inflammatory effect in multiple sclerosis (MS). Testosterone has an effect in protecting neurons in culture against glutamate-induced toxicity and oxidative stress, and it stimulates myelin formation and regeneration mediated through the neural androgen receptor (AR). Testosterone treatment has potential neuroprotective effects in men with relapsing-remitting MS. Testosterone induces anti-inflammatory as well as neuroprotective effects, and transdermal testosterone in male MS patients appears promising.
Systemic Lupus Erythematosus	Pakpoor et al., 2018 [84] Gordon et al., 2008 [85] Sasaki et al., 2006 [86] Olsen & Kovacs, 1995 [87] Bizzarro et al., 1987 [88] Amor et al., 1983 [89] Costello & Singer, 1952 [90]	Lupus erythematosus, chronic disseminated type, showing a dramatic response to testosterone therapy. Androgenic steroids can exert significant effects on immune parameters, and this suggests that the effects of androgens on the immune system may contribute to the sexual dimorphism of autoimmune diseases. Treatment with a high dose of methyltestosterone improved thrombocytopenia and symptoms, suggesting that methyltestosterone may have a clinical benefit in the treatment of patients with Klinefelter’s syndrome with a low level of testosterone accompanying immunological disorders. The positive association between testicular testosterone deficiency and systemic lupus erythematosus (SLE) supports the hypothesis that low testosterone levels may influence the development of male SLE. Of clinical importance, males with SLE are at increased risk of co-morbid testicular hypotestosteronemia, and this may warrant consideration in the management of patients with testosterone therapy.

## Abbreviations

C-reactive protein	CRP
Tumor necrosis factor-α	TNF-α
Interleukin-1β	IL-1β
Interleukin-6	IL-6
Interleukin-10	IL-10
Non-small cell lung cancer	NSCLC
High-density lipoprotein	HDL
Coronary artery disease	CAD
Psoriasis area and reduced severity index	PASI
Physician global assessment for psoriasis	PGA
Androgen receptor	AR
5α-dihydrotestosterone	5α-DHT
Group 2 innate lymphoid cells	ILC2
5-lipoxygenase	5-LO
Type 2 extracellular receptor kinase	ERK2
Chronic obstructive pulmonary disease	COPD
Experimental autoimmune encephalomyelitis	EAE
Interferon-γ	IFN-γ
Multiple sclerosis	MS
Blood peripheral mononuclear cells	PBMCs
Tumor growth factor β1	TGFβ1
Natural killer cells	NK
Brain-derived neurotrophic factor	BDNF
Platelet-derived growth factor	PDGF-BB
Systemic lupus erythematosus	SLE
Pathogen-associated molecular patterns	PAMPs
Damage-associated molecular patterns	DAMPs
Toll-like receptors	TLRs
Nuclear factor kappa-light-chain-enhancer	
of activated B cells	NF-κB
Lipopolysaccharides	LPS
Inducible nitric oxide synthase	iNOS
Inducible nitric oxide	iNO
Experimental autoimmune orchitis	EAO
Androgen receptor knockout mouse model	ARKO
Monocyte chemoattractant protein-1	MCP-1
Receptor for activated C kinase 1	RACK1
